# A Single Crystal Process Window for Electron Beam Powder Bed Fusion Additive Manufacturing of a CMSX-4 Type Ni-Based Superalloy

**DOI:** 10.3390/ma14143785

**Published:** 2021-07-06

**Authors:** Julian Pistor, Christoph Breuning, Carolin Körner

**Affiliations:** 1 Joint Institute of Advanced Materials and Processes, Friedrich-Alexander Universität Erlangen-Nürnberg, 90762 Fürth, Germany; carolin.koerner@fau.de; 2 Department of Materials Science and Engineering, Chair of Materials Science and Engineering for Metals, Friedrich-Alexander Universität Erlangen-Nürnberg, 91058 Erlangen, Germany; christoph.breuning@fau.de

**Keywords:** electron beam melting, Ni-based superalloy, single crystal, grain selection, microstructure

## Abstract

Using suitable scanning strategies, even single crystals can emerge from powder during additive manufacturing. In this paper, a full microstructure map for additive manufacturing of technical single crystals is presented using the conventional single crystal Ni-based superalloy CMSX-4. The correlation between process parameters, melt pool size and shape, as well as single crystal fraction, is investigated through a high number of experiments supported by numerical simulations. Based on these results, a strategy for the fabrication of high fraction single crystals in powder bed fusion additive manufacturing is deduced.

## 1. Introduction

Single crystal Ni-based superalloys are state-of-the-art materials for high-temperature applications in turbine blades at both high temperatures and high mechanical loads. Due to their lack of high angle grain boundaries and the highly optimized microstructure, through alloying with up to 10 alloying elements, they can withstand temperatures up to 1100 °C in long-term exposure [1]. Typical single crystal alloys, such as CMSX-4 developed by Cannon Muskegon, are optimized for casting processes according to the Bridgman process [2,3]. In this case, the material is cast into a ceramic shell mold and then directionally solidified by applying a well-defined temperature gradient and solidification velocity [4,5]. In addition, a so-called grain selector helix is introduced in the shell mold to select only one well-oriented grain of the initial columnar grain structure [6]. However, this production route incorporates some major drawbacks like the lack of part complexity and the high amount of residual elemental segregation, leading to expensive and time-consuming post-processing [7]. Therefore, several groups already have investigated this type of non-weldable casting alloys by powder-based additive manufacturing [8,9,10,11,12,13,14], where elemental segregation is much smaller due to high solidification rates [15]. Since these alloys are not optimized for additive manufacturing, processing is challenging. Specifically, the high amount of γ’ precipitates (up to 80 vol.%) and the strong tendency for segregation lead to a high susceptibility for cracks including solidification cracking [16], liquation cracking [17] or strain age cracking [18,19]. Recent studies have shown that apart from alloy modifications [20,21,22], either a high amount of high angle grain boundaries or no high angle grain boundaries at all (single crystalline, SX) are necessary to process these alloys without cracks [8,23].

The first successful build of CMSX-4 single crystals via electron beam powder bed fusion (E-PBF) was reported in 2016 by Ramsperger et al. [24]. Other researchers proved the possibility of building single crystals by processing weldable [25] or non-weldable [8] Ni-based superalloys with E-PBF. In all cases, grain selection from a columnar to a single crystalline microstructure was achieved by a specific well-defined scanning strategy without using a single crystalline substrate. As expected, elemental segregation was strongly reduced due to the high solidification rate inherent to additive manufacturing [15,26]. This leads to a strong reduction of post-processing time (heat treatment) [27] and also improved performance. Investigations of high-temperature phase stability [26], low cycle fatigue [28], high-temperature strength as well as creep [29] showed the beneficial effect of the additive manufacturing processing route in comparison to the conventional casting one. However, there is an important drawback of additively manufactured single crystals. There is always a polycrystalline shell with varying thickness. Shell formation can be traced back to the thermal conditions at the surface of the component [30] but was, up to now, not very well understood.

The main goal of this work is to elucidate the necessary prerequisites for single crystal formation during additive manufacturing. For this, the interplay between the processing parameters and the resulting microstructure is investigated in detail. In addition, the experimental work is accompanied by numerical simulation to correlate microstructure evolution with the thermal conditions at the solidification front. From these considerations, the requirements for single crystal evolution are revealed. Based on this, a strategy for minimizing the polycrystalline shell is presented.

## 2. Materials and Methods

**Figure 1:**
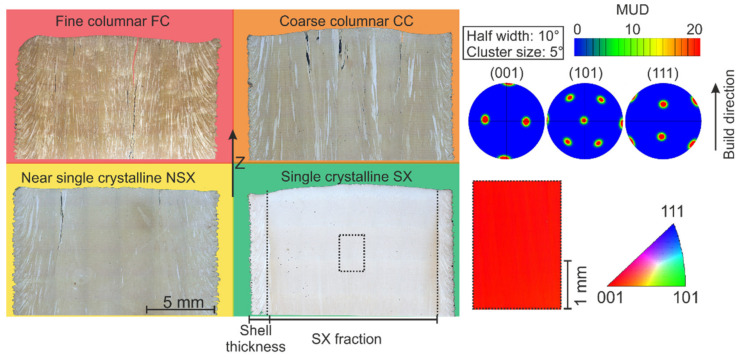
Microstructure classification: Fine columnar (FC), coarse columnar (CC), near single crystalline (NSX) and single crystalline (SX). The dashed rectangular area marks the area of the EBSD measurement, to verify the present single crystal microstructure.

The CMSX-4 powder used for sample preparation was provided by TLS Technik GmbH & Co. Spezialpulver KG (Bitterfeld-Wolfen, Germany). The powder was atomized using argon gas and CMSX-4 rods as feedstock. The chemical composition of the powder lies within the specification of the CMSX-4 alloy [29]. Powder shape and size distribution were characterized by light microscopy (Axio M1m Imager, Carl Zeiss AG, Oberkochen, Germany) and laser diffraction (Mastersizer 3000, Malvern Panalytical GmbH, Kassel, Germany). Besides the spherical powder particles, only a small quantity of satellites was present. The gas-atomized powder showed a particle size distribution of 45–105 μm with D_10_ = 44.8 μm, D_50_ = 70.7 μm and D_90_ = 102.0 μm. The powder was processed in an Arcam A2 machine operating at 60 kV accelerating voltage under a controlled atmosphere of 2 × 10^−3^ mbar helium pressure. More information about the setup and E-PBF process can be found in [23,24]. Here, the operating temperature was 1030 °C. The temperature was kept constant by raster scanning the powder bed with the defocused electron beam. The layer thickness (50 μm) and the scanning pattern (snake-like scan pattern with line order 1 and 90° rotation after each layer, i.e., cross snake 90°) were constant. Contouring was not applied. Each build process consisted of 12 cuboids with 15 × 15 × 25 mm^3^ dimensions. Beam velocity v_Beam, beam power P and hatching distance l_0_ were varied. The lateral beam speed v_lateral given by v_lateral = v_Beam l_0_/15 mm and the area energy E_A_ = P/(l_0_ v_Beam) are used as parameters. Prior to the metallographic preparation, laser scanning microscopy (LSM—Lext Ols 4000, Olympus Europa SE & CO. KG, Hamburg, Germany) was applied to analyze the surface of chosen samples. Cross-sections parallel to the build direction of the cuboids were prepared for the determination of the melt pool depth, melt pool lateral extension and the grain structure in the center region. The cut along the build direction was perpendicular to the scanning vector of the last layer (i.e., the cutting direction was the lateral beam movement direction during melting of the last layer). Metallographic preparation of the samples was performed in a standard manner by means of grinding with SiC and two-step polishing with 3 μm diamond particles and OPU suspension as well as etching with V2A stain (mixture of HCl, HNO_3_, H_2_O and Vogel’s special reagent) at 60 °C, to reveal the grain structure. For a qualitative classification of the grain structure in the center region of the samples, four microstructure types rated from 0 to 3 were introduced, see Figure 1. The higher the rate, the closer is the microstructure to a single crystal (SX = 3). For this purpose, light microscopic images were used (Axio M1m Imager, Carl Zeiss AG, Oberkochen, Germany). In addition, electron backscatter diffraction (EBSD) measurements using a Helios NanoLab 600 FIB/SEM (FEI Company, Hillsboro, OR, USA) with a step size of 5 μm were performed, to verify the single crystalline microstructure.

The single crystal grain structure (SX) is characterized by a core region, which is homogeneous in both primary and secondary dendritic orientation. The near-SX microstructure (NSX) is close to the single crystal one, but with very few columnar stray grains oriented along the build direction. The coarse columnar (CC) and fine columnar structures (FC) show columnar grains oriented in the build direction. The applied rating system to describe the expected grain structures in dependency of the process parameters with the different colors is based on SX = 3, NSX = 2, CC = 1 and FC = 0.

**Figure 2:**
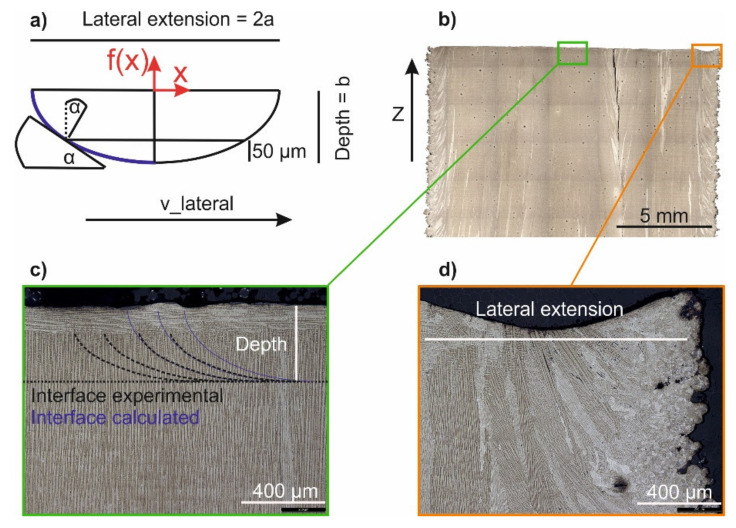
Melt pool characterization. (**a**): Thermal gradient at the solidification front is characterized by the angle α. (**b**): Overview image. (**c**): Determination of the melt pool depth based on experimental and analytically calculated rest-lines. (**d**): Determination of the melt pool lateral extension.

The cross-sections parallel to the build direction were used to determine melt pool depth (b) and lateral extension (2a) according to Figure 2b–d. Only the rear part of the melt pool (blue) is considered for the analytical calculation of the solid-liquid interface, as only here solidification takes place (Figure 2a).

Experiments have shown that the rear side of the melt pool can be well-approximated by using an elliptical Equation (1) with the melt pool depth b and half the lateral extension a as variables:

(1) $$f\left(x\right)=-\frac{b}{a}\sqrt{a^{2}-x^{2}}$$

Individual melt pools appear in form of rest-lines in the top melt layer. The solidification direction, characterized by the angle α, is perpendicular to the rest-lines. From the tangent slope (Equation (2)), α can be calculated according to Equation (3).

(2) $$\frac{\partial f\left(x\right)}{\partial x}=\frac{\mathrm{bx}}{a}\frac{\;1}{\sqrt{a^{2}-x^{2}}}$$

(3) $$\alpha =\tan^{-1}\left(\frac{\partial f\left(x\right)}{\partial x}\right)\;$$

For 50 μm layers, only 50 μm above the pool depth are relevant for grain structure formation. Everything else is remelted during the next layer. The angle *α* characterizes the solidification direction of the material with respect to the build direction, and increases from the melt pool bottom with 0° to the calculated value of α after f(x) reaches −b + 50 μm due to solidification. The respective value for x can then also be determined with Equation (1).

A semi-analytical heat conduction model was used to compute and classify the melt-pool geometries. The thermal model is based on an analytical solution for the transient temperature response to a moving volumetric Gaussian heat source (Equation (4)) [31,32]. The temperature T at time t is defined by:

(4) $$T-T_{0}=\frac{2\eta P}{\rho c{\left(\pi /3\right)}^{3/2}}\int_{o}^{t}\frac{1}{\sqrt{\phi_{x}\phi_{y}\phi_{z}}}\;\exp\left(\frac{-3x{\left(t\prime \right)}^{2}}{\phi_{x}}+\frac{-3y{\left(\;t\;\prime \right)}^{2}}{\phi_{y}}+\frac{-3z{\left(t\prime \right)}^{2}}{\phi_{z}}\right)\mathrm{dt}\prime$$

where T_0_ is the preheating temperature, η is the absorption coefficient, P is the beam power, ρ is the density, c the specific heat, and

(5) $$\phi_{i}=12\beta \left(t-t\prime \right)+\varphi_{i}{}^{2},\;\mathrm{for}\;i=x,y,z$$

where the volumetric Gaussian beam shape (Equation (5)) is defined in each dimension by a beam width $\varphi_{i}$ and the thermal diffusivity β. The heat source motion is described by the coordinate system, where x, y and z describe the distance from the point of interest to the transient location of the beam at time t ′. The piece-wise definition of the scan path prohibits the analytical integration of Equation (4), so a Gaussian quadrature scheme is used to numerically integrate the temperature at a given time and location [33]. The model neglects effects of fluid convection, latent heat release, radiation and vaporization. Material properties are assumed to be constant and uniform. The model is calibrated by adjusting the preheating Temperature $T_{0}$ to match the analytical and experimental melt pool depth of a standard parameter set.

## 3. Results

**Figure 3:**
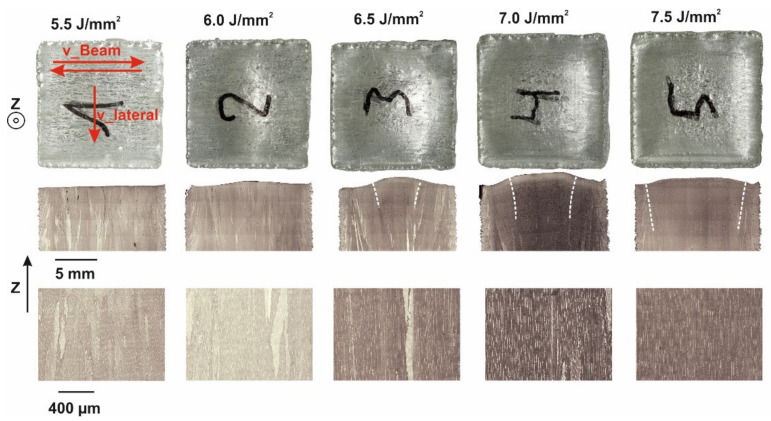
Influence of the energy input at constant v_lateral. Top: Surface topography. Middle: Light microscopic length sections showing the microstructure and bulges at the surface. The dashed white line indicates the SX region. Bottom: Magnification of the microstructure. (v_Beam: 400 mm/s, hatch distance: 100 μm, v_lateral: 2.67 mm/s).

Figure 3 shows the influence of the energy input from 5.5 up to 7.5 J/mm^2^ at constant lateral speed v_lateral on the resulting grain structure. The microstructure changes continuously from fine columnar (FC) to single crystalline (SX) with increasing energy input. Starting with an FC microstructure for 5.5 J/mm^2^ the center of the sample begins to coarsen with increasing energy input. The transition from coarse columnar (CC) to near SX happens in a very narrow energy regime between 6 to 6.5 J/mm^2^. By further increasing the energy input from 6.5 to 7.5 J/mm^2^, a single crystalline area appears in the center, which expands over the sample until only the shell remains polycrystalline.

**Figure 4:**
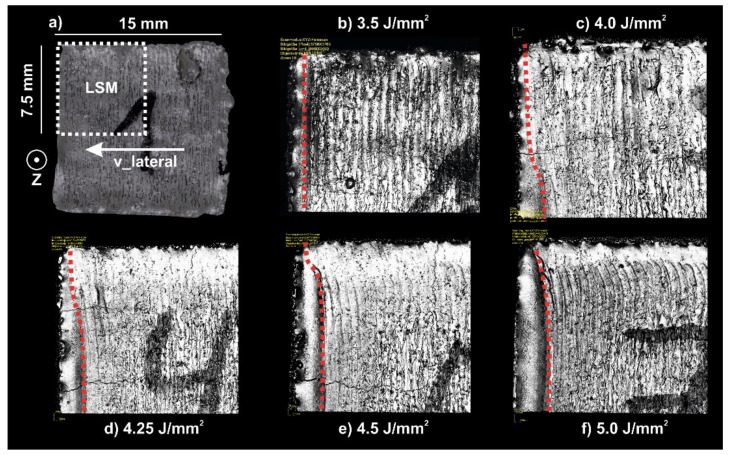
Influence of the energy input on the surface topography at a constant v_lateral. Lateral movement was from right to left. (**a**): schematic drawing of the LSM measurement position. (**b**–**f**): LSM images of the development of residual melt pool shape with increasing energy input. (v_Beam: 400 mm/s, hatch distance: 200 μm, v_lateral: 5.33 mm/s).

At the sample surface (photographic images in the top row of Figure 3), the residual melt pool is visible. For this v_lateral of 2.67 mm/s at low energy input (5.5 J/mm^2^), straight melt tracks according to the scan vectors can be observed. With increasing energy input (6.0 J/mm^2^), the melt tracks start to bend along the scanning direction and a small ditch, the residual melt pool, at the end of the melted layer emerges. The extension of this ditch increases with increasing energy input (6.5–7.0 J/mm^2^) in the scanning direction until it reaches nearly the sample width of 15 mm at 7.5 J/mm^2^. Obviously, the evolution of the melt pool shape and the appearance of an SX core are correlated to each other. The residual melt pool shape and the surface topology according to laser scanning microscopy (LSM) as a function of energy input at v_lateral = 5.33 mm/s are depicted in Figure 4 in more detail. By increasing the energy input from Figure 4b–f, the evolution of the residual melt pool shape indicated by red dashed lines becomes clear. Once the energy input is sufficiently high (Figure 4c), the center areas are kept as liquid, until the beam returns and the melt pool persists in these regions (persistent melt pool). This evolution of the melt pool is accompanied by changes in the sample topography. Whereas the surface of the sample in Figure 4b is completely flat, sample Figure 4f is uneven at high energies due to the ditch correlated with the persistent melt pool.

**Figure 5:**
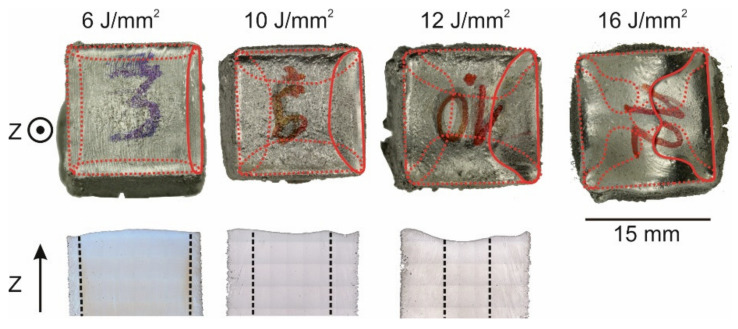
Effect of the energy input beyond the persistent melt pool threshold. Top: Residual melt pool at the surface (red solid line: last melt pool, red dashed line: melt pool from other hatching directions). Bottom: Microstructure with SX core indicated by black dashed lines (the 16 J/mm^2^ sample was unstable). (v_Beam: 400 mm/s, hatch distance: 150 μm, v_lateral: 4 mm/s).

By further increasing the energy input, a strong extension of the residual melt pool is observed, see Figure 5. Here the melt pool contour is marked by a red solid line, whereas the dashed lines represent the melt pool in previous layers, whose hatch direction was rotated by 90° with respect to the next layer. The increasing energy input leads to swelling of the surface that causes process instabilities. Hence, the specimen produced using 16 J/mm^2^ needed to be stopped during the build process, and the grain structure could not be evaluated. However, the surface of the 16 J/mm^2^ sample was used to understand the growth behavior of the persistent melt pool at such high energies. All samples of Figure 5 show a single crystalline core according to light microscopic images with SX fraction from 80% at 6 J/mm^2^ to 35% at 12 J/mm^2^.

**Figure 6:**
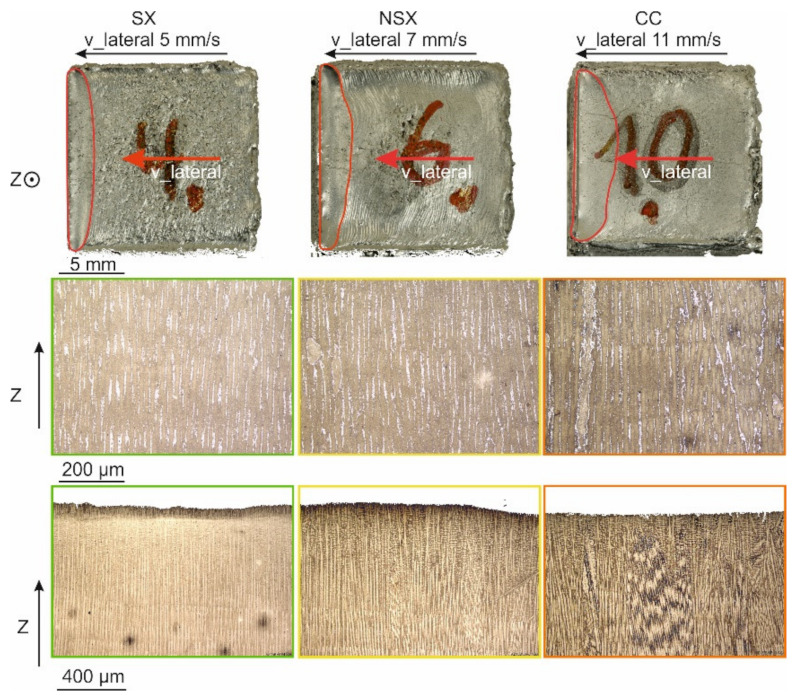
Effect of increasing v_lateral beyond the persistent melt pool threshold at a constant energy input of 6 J/mm^2^ and 100 μm hatch distance. Top: Sample surface with residual melt pool. Middle: Microstructure of the sample centers. Bottom: Microstructure of the top layers.

The influence of the v_lateral at constant energy input is depicted in Figure 6. The starting point is the SX regime with an area energy of 6 J/mm^2^. Increasing the v_lateral leads to a transition from single crystalline to near SX and eventually to columnar microstructures (Figure 6 middle row). Parallel, the melt pool changes from cigar-like with nearly parallel flanks to a very broad shape as indicated by the red line on the photographic images in the top row of Figure 6. The higher the v_lateral, the larger is the lateral extension of the melt pool. The light microscopy of the center part and the top layers (Figure 6 middle and bottom row) shows that the primary dendrite spacing slightly increases from 12 to 16 μm, when enhancing v_lateral from 5 to 11 mm/s. In addition, new grain formation takes place for the high velocities, especially at the melt pool bottom, and misorientations between the build direction and the primary dendrites emerge, see Figure 6 middle and bottom row.

**Figure 7:**
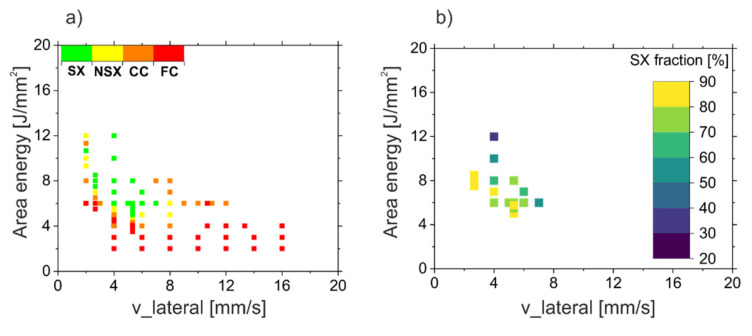
SX process window. (**a**): Microstructure map in dependency of area energy and v_lateral for the investigated hatch distances between 100 to 200 μm, (**b**): SX-fraction of the samples of (**a**) determined according to Figure 1.

Figure 7 shows the process window for SX evolution, which summarizes the experimental findings. The different regions are classified according to fine columnar (FC), coarse columnar (CC), near single crystalline (NSX) and single crystalline (SX) (Figure 7a).

Obviously, different combinations of area energy and v_lateral lead to an SX core. The minimum energy input for the SX regime decreases with increasing velocity. The SX process window is surrounded by columnar structures to lower energies as well as to higher velocities. A change of the process parameters leads to a continuous transformation from a single crystal to near single crystalline and eventually to columnar structures, since more and more columnar grains emerge. However, in the investigated regime, there appears to be not much change in the microstructure caused by increasing the energy input in the SX window. For all SX samples presented in Figure 7a, the single crystal fraction was determined according to the procedure presented in Figure 1 by using the measured SX width and the specimen width, see Figure 7b. This shows that the SX fraction changes by varying the process parameters. The highest SX fractions, over 80%, are achieved at the lower boundary of the SX process window. By increasing the energy input or the v_lateral, the SX fraction decreases.

**Figure 8:**
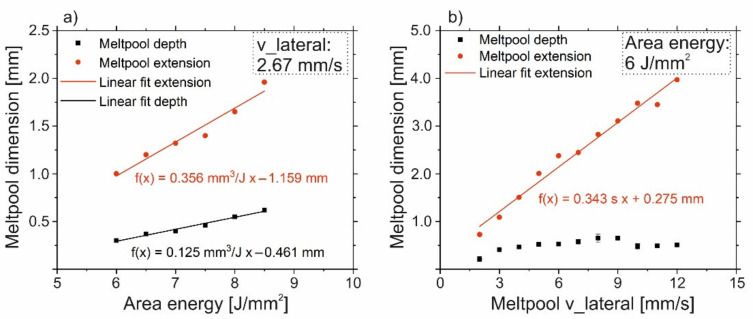
Melt pool dimension. (**a**): Influence of an increasing energy input at a given v_lateral of 2.67 mm/s on the melt pool lateral extension and depth (Figure 3). (**b**): Influence of the v_lateral at a given energy input of 6 J/mm^2^ on the melt pool lateral extension and depth (Figure 6).

To understand the transitions from columnar structures to single crystals and vice versa, the melt pool shape was investigated in detail. Figure 8a shows the melt pool depth and lateral extension for increasing area energy at constant v_Beam and hatch distance. Both, the melt pool depth as well as the lateral extension increase in a linear way, while the ratio between lateral extension and depth is about three and more or less constant for this v_lateral of 2.67 mm/s. The extrapolation of both lines to f(x) = 0 indicates a minimum energy input for the melting of the material at this v_lateral between 3.2 and 3.7 J/mm^2^. The results are different for increasing v_lateral at constant area energy and hatch distance, see Figure 8b. For low velocities, the melt pool depth increases with increasing velocity. At higher velocities, the melt pool depth approaches a constant value given by the applied area energy. The melt pool lateral extension increases with increasing velocity in a linear way. Thus, the ratio between lateral extension and depth depends on the v_lateral and is between 3 and 8. This means the shape of the melt pool is very sensitive to the velocity, whereas the influence of area energy is low.

## 4. Discussion

In the following section, the solidification conditions dependent on the melt pool shape are correlated with the resulting microstructure. The first important experimental observation is that an SX core only develops in the presence of a persistent melt pool, i.e., a permanent melt pool along the hatching direction. SX microstructures do not appear as long as a trailing melt pool is present, where the melt pool follows the movement of the beam.

**Figure 9:**
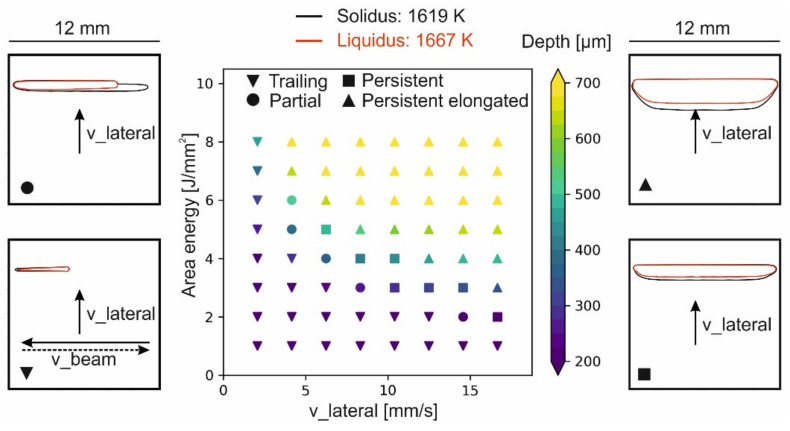
Simulation of the melt depth and the appearance of the melt pool as a function of the v_lateral and the area energy for a hatch distance of 100 μm. Beam scanning direction and lateral movement as indicated.

Figure 9 shows a numerically calculated map for the stationary melt pool geometry as a function of the v_lateral and the area energy. Low velocities or energies result in a trailing melt pool. Increasing the velocity or the area energy leads to a partial and eventually to a fully persistent melt pool. A partially persistent melt pool at the transition from a trailing to a fully persistent melt pool can also be experimentally observed in Figure 4. The persistent melt pool starts from the middle of the scanning vector and expands in direction of the turning points with increasing area energy. With increasing beam velocity, the energy necessary for a fully persistent melt pool decreases, since the beam return time, $t_{\mathrm{return}}=l_{\mathrm{return}}/v\_\mathrm{Beam}$, decreases. The return length $l_{\mathrm{return}}$ is the length until the beam returns to a specific position of the scanning vector. Thus, lower return times reduce thermal losses due to diffusion, and the energy threshold for a persistent melt pool decreases. 

**Figure 10:**
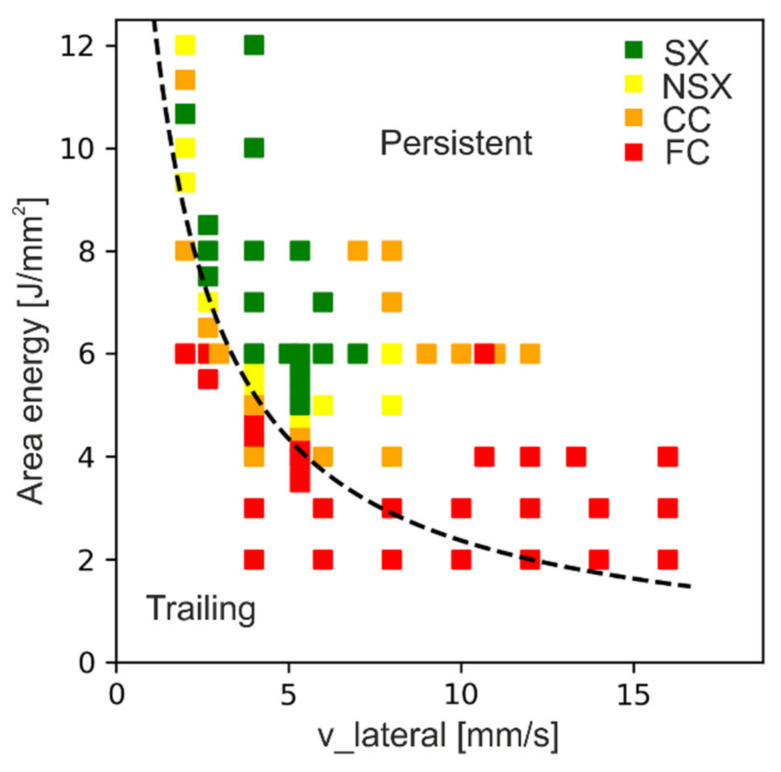
Experimental microstructure data from Figure 7(**a**) and calculated separation line from Figure 9 between trailing melt pool and persistent melt pool.

Further increase in the beam velocity or area energy leads to a widening of the melt pool. Comparison of the numerical results of Figure 9 with Figure 7 confirms the experimental observation that the appearance of SX or NSX microstructure is closely related to the presence of a persistent melt pool, see Figure 10. The persistent melt pool leads to defined solidification conditions, which are necessary for SX formation. Nevertheless, the presence of a persistent melt pool is necessary but obviously not sufficient for the evolution of an SX microstructure.

**Figure 11:**
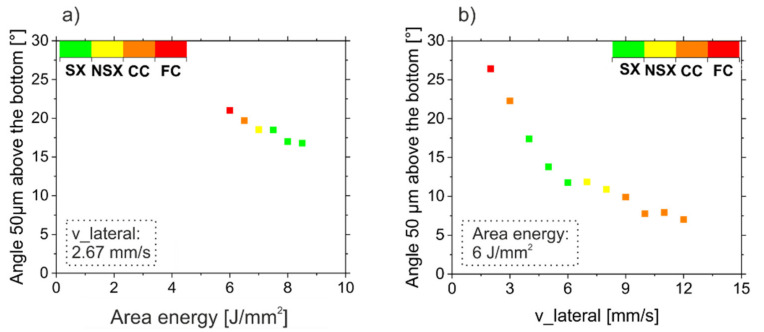
Microstructure as a function of the solidification conditions for a hatch distance of 100 μm according to analytical calculation. (**a**): Solidification angle α as a function of the area energy at constant v_lateral of 2.67 mm/s (samples of Figure 3 and Figure 8 (a)). (**b**): Solidification angle α as a function of the v_lateral at constant area energy of 6 J/mm^2^ (samples of Figure 6 and Figure 8(b)).

The solidification direction was investigated in order to characterize the solidification conditions at the melt pool in more detail. For this, the maximum angle α of the thermal gradient (perpendicular to the solid-liquid interface) with respect to the build direction was analytically determined based on Equation (3) for all samples shown in Figure 8. The results are depicted in Figure 11. It is important to note that the maximal angle α in the relevant region, which is not remelted in the subsequent layer, appears at 50 μm (50 μm = layer thickness) height above the melt pool bottom. The angle α decreases with increasing area energy input since the melt pool depth increases, see Figure 9 and Figure 11a, while the shape is more or less constant. Lower angles cannot be realized for this parameter set, since the high energy input leads to surface bulges. Increasing the velocity at constant energy input, leads to a pronounced reduction of α due to the strong increase in the melt pool lateral extension (Figure 11b). In addition, the resulting microstructure is specified in Figure 11 using different colors. If α is too high or too low, a columnar microstructure develops. A single crystalline microstructure developed for α between 12° and 17°.

The role of the solidification angle between successive layers for tailoring the grain structure was already reported by Körner et al. [34], Helmer et al. [35] and Gotterbarm et al. [25] for IN718 for individual samples. Fine columnar (FC) microstructures only appear for high angles (>20°), where grain selection and coarsening are balanced by new grain formation [34]. Even higher angles (>30°) may lead to equiaxed microstructures [35]. Reducing the solidification angle also reduces the new grain density and thus leads to coarse columnar (CC) microstructures. These CC microstructures evolve into near single crystalline (NSX) or single crystalline (SX) microstructures for angles between around 12° and 17°. For angles below this region, again CC microstructures emerge, as primary dendrites with an angle deviation bigger than the respective criteria for small-angle grain boundaries emerge [11,36]. 

**Figure 12:**
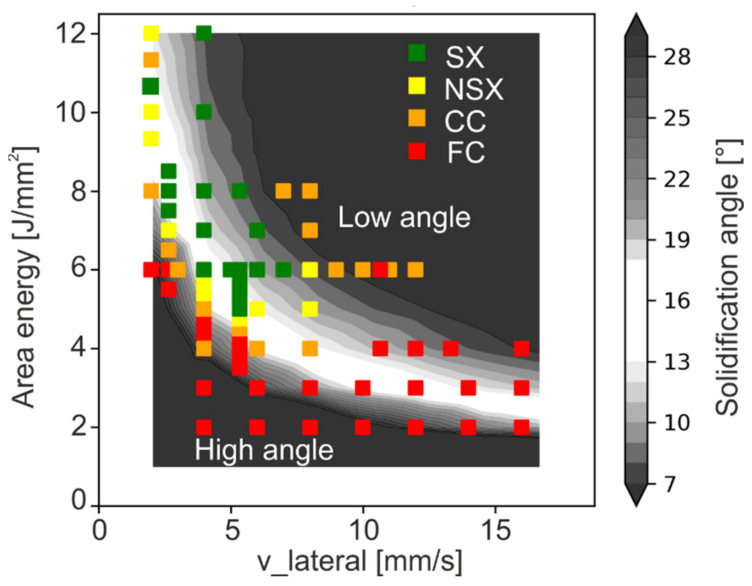
Microstructure map with calculated solidification angle α. Light colors represent regions, where the melt conditions are expected to enable the formation of NSX or SX microstructures according to numerical simulation.

Figure 12 sums up the correlation between the experimentally observed microstructure (Figure 7) and the respective numerically calculated solidification angle. Here bright areas indicate regions where SX microstructures are expected based on the present solidification angle. The before-mentioned relationships seem to hold true for v_lateral up to around 7 mm/s. However, with increasing v_lateral, the formation of SX or NSX structures is disrupted, even if the solidification angle is adjusted properly. Therefore, there is more to consider than a present persistent melt pool and the proper solidification angle. An explanation for this transition can be derived from the analysis of the melt pool depth. As indicated by the numerical simulation in Figure 9, the melt pool depth of the SX area is at least at around 500 μm. At higher v_lateral the melt pool depth for the required solidification angle is strongly reduced and a transition to columnar structures occurs for 8 mm/s and 4 J/mm^2^ with a present melt pool depth of around 400 μm (see Figure 9andFigure 12). This reduction of the melt pool depth may lead to increased statistical fluctuations of the solidification conditions in the lowest 50 μm, when keeping the layer thickness constant. Those may be either fluctuations in solidification angle or fluctuations in thermal gradient and solidification velocity. Both of them are expected to influence the grain structure evolution and are part of ongoing research and therefore still under investigation.

**Figure 13:**
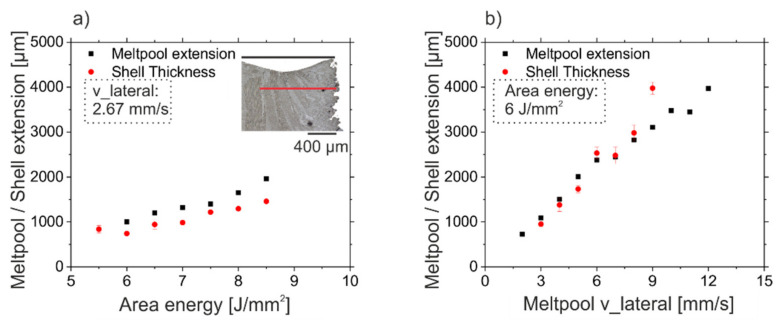
Correlation between shell thickness and lateral extension of the melt pool. (**a**): Shell thickness and melt pool lateral extension as a function of the area energy at constant v_lateral of 2.67 mm/s (Specimens of Figure 3). (**b**): Shell thickness and melt pool extension as a function of the v_lateral at constant energy input of 6 J/mm^2^ (Specimens of Figure 6).

Even if an SX core evolves, the SX fraction of the samples is strongly different between different parameter sets, see Figure 7b. With increasing v_lateral or for increasing area energy input, the extension of the polycrystalline shell area increases, whereas the core region, regardless of the microstructure, decreases. Figure 13a shows the dependency of the area energy at constant v_lateral. There is a clear correlation between the lateral extension of the persistent melt pool and the shell thickness. Figure 13b shows the lateral extension of the persistent melt pool and the shell thickness as a function of the v_lateral at constant area energy. The single crystal core region decreases with higher energy input and higher v_lateral (see Figure 7b) due to the increasing lateral extension of the persistent melt pool. Thus, the highest SX fractions of 85% in this study were achieved at the lowest possible combinations of energy input and v_lateral in the SX process window. A further reduction of the energy input or the v_lateral is not possible, since the melt pool is no longer persistent.

## 5. Conclusions

A process map for the microstructural evolution of single crystals during electron beam powder bed fusion additive manufacturing was experimentally determined. Different combinations of area energy and v_lateral lead to SX microstructures. At constant area energy, the microstructure develops with increasing v_lateral from fine columnar, to coarse columnar, to nearly SX and eventually to SX. Further increase in the velocity reduces the SX fraction and finally leads again to columnar microstructures. At constant v_lateral and increasing area energy, the microstructure turns again from fine columnar, to coarse columnar, to nearly SX and eventually SX. However, in the investigated regime, a further increase in the energy input does not lead to a change in the microstructure of the center. Analysis of the melt pool geometry combined with numerical simulation lead to the following conclusions:

1. The formation of a persistent melt pool is a necessary prerequisite for grain selection to gain a single crystal. The persistent melt pool guarantees defined solidification conditions within one layer.
2. The maximum tilt angle α of the thermal gradient during solidification with respect to the build direction can be connected to the resulting microstructure. NSX or SX microstructures developed between around 12° and 17° applying hatch distances between 100 and 200 μm and lateral speeds between 2 up to 7 mm/s. At higher lateral speeds, SX formation is disrupted, even if the solidification angle is adjusted properly.
3. The lateral extension of the persistent melt pool determines the thickness of the polycrystalline shell. With increasing energy input at constant velocity, the shape of the persistent melt pool is preserved, i.e., lateral extension and melt pool depth grow in the same manner. On the other hand, at constant energy input, the lateral extension increases linearly with increasing v_lateral, whereas the melt pool depth approaches a constant value given by the energy input.

In conclusion, samples with the highest SX fraction emerge at the lowest possible combination of energy input and v_lateral, where a fully persistent melt pool with proper solidification angle and melt pool depth is present.

## Data Availability

Data sharing not applicable.
